# Postnatal Overfeeding Causes Early Shifts in Gene Expression in the Heart and Long-Term Alterations in Cardiometabolic and Oxidative Parameters

**DOI:** 10.1371/journal.pone.0056981

**Published:** 2013-02-26

**Authors:** Ahmed Habbout, Charles Guenancia, Julie Lorin, Eve Rigal, Céline Fassot, Luc Rochette, Catherine Vergely

**Affiliations:** 1 Inserm UMR866, Laboratoire de Physiopathologie et Pharmacologie Cardio-Métaboliques (LPPCM), Faculties of Medicine and Pharmacy, University of Burgundy, Dijon, France; 2 Inserm UMR1083, Biologie neurovasculaire et mitochondriale intégrée, Angers, France; University of Western Ontario, Canada

## Abstract

**Background:**

Postnatal overfeeding (OF) in rodents induces a permanent moderate increase in body weight in adulthood. However, the repercussions of postnatal OF on cardiac gene expression, cardiac metabolism and nitro-oxidative stress are less well known.

**Methodology/Principal Findings:**

Immediately after birth, litters of C57BL/6 mice were either maintained at 10 (normal-fed group, NF), or reduced to 3 in order to induce OF. At weaning, mice of both groups received a standard diet. The cardiac gene expression profile was determined at weaning and cardiac metabolism and oxidative stress were assessed at 7 months. The cardiac expression of several genes, including members of the extracellular matrix and apelin pathway, was modified in juvenile OF mice. In adult mice, OF led to an increase in body weight (+30%) and to significant increases in plasma cholesterol, insulin and leptin levels. Myocardial oxidative stress, SOD and catalase activity and mRNA expression were increased in OF mice. *In vivo*, diastolic and systolic blood pressures were significantly higher and LV shortening and ejection fraction were decreased in OF mice. *Ex vivo*, after 30 min of ischemia, hearts isolated from OF mice showed lower functional recovery and larger infarct size (31% vs. 54%, p<0.05). Increases in collagen deposition and expression/activity of matrix-metalloproteinase-2 were observed in adult OF mouse hearts. Moreover, an increase in the expression of SOCS-3 and a decrease in STAT-3 phosphorylation were observed in ventricular tissues from OF mice.

**Conclusions/Significance:**

Our study emphasizes that over-nutrition during the immediate postnatal period in mice leads to early changes in cardiac gene expression, which may permanently modify the heart’s structural organization and metabolism and could contribute to a greater susceptibility to myocardial ischemia-reperfusion injury.

## Introduction

Clinical and experimental evidence indicates that the environment during the peri-natal period and early development plays a key role in regulating metabolic tendencies in adulthood, and that nutrition in early life has an impact on the subsequent risk of overweight, hypertension and insulin resistance. Indeed, due to plasticity in gene expression, the neonatal period is critical for the orientation of phenotypic features in adulthood, since epigenetic modifications may permanently impact gene expression and influence the development of chronic diseases. In this field, several groups have shown that inducing early post-natal over-nutrition in rats, just by reducing litter sizes, led to substantial changes in body weight at weaning (nearly 30% increase), which persisted at a lower level in mature animals (10–15% increase) [1]–[3]. In recent research at our laboratory [4] we showed that a cluster of symptoms characteristic of metabolic syndrome occurred in neonatally overfed rats. This finding has led to clearer understanding of the cardiovascular and metabolic consequences of this overweight in rats. We also showed that the cardiac redox environment was modified in overweight adult rats.

Genetically modified mice are of great value to investigate the role of specific genes in the development of diseases. While much work has been done on the cardio-metabolic consequences of genetically db/db or ob/ob obese mice or of high-fat diets, little research has been done on the cardiovascular consequences of simple moderate overweight [5]. In this field, postnatal overfeeding might provide a good experimental model to study the classical risk factors of cardiovascular disease associated with early-induced permanent overweigh. However, very few studies in mice have evaluated the impact of post-natal overfeeding induced by litter reduction on cardio-metabolic and oxidative status in adulthood.

Therefore, the aim of our work was to evaluate the metabolic, oxidative and cardiovascular consequences of moderate overweight induced by post-natal over-nutrition in adult C57BL/6 mice. Additionally, gene expression in the hearts of juvenile (post-weaning) overfed mice was compared with that of normally fed mice in order to determine early changes in gene expression that may be involved in subsequent differences in myocardial function.

## Materials and Methods

### Animals

Adult female C57BL/6 mice (Charles River, L’Arbresle, France) were caged with male mice at a proportion of 2∶1, then housed in individual cages and given water and a standard pellet diet *ad libitum* during pregnancy and lactation. On the second day of life, male pups were randomly distributed among the mothers to achieve cross-fostering and the litter size was adjusted to 10 male pups (normal-fed, NF group), or reduced to 3 male pups in order to induce postnatal overfeeding (over-fed, OF group). Excess pups were rapidly killed by decapitation after anesthesia with isoflurane. After weaning (day 24), mice of both groups had free access to a standard diet (A03, SAFE Diets Augy, France) and water. Throughout life, body weight and food intake were measured weekly then monthly. All animals received humane care and study protocols complied with the institution’s guidelines. The investigation conformed to Directive 2010/63/EU of the European Parliament and to the *Guide for the Care and Use of Laboratory Animals* published by the US National Institutes of Health (NIH Publication No. 85–23, revised 1996) and was approved by the local ethics committee (Comité d’Ethique de l’Expérimentation Animale, Université de Bourgogne, Dijon, France, protocol agreement number: 3710). Throughout the procedure, care was taken to avoid suffering and to ensure animal welfare, for instance through enrichment of the environment in cages.

### Body Composition

Whole body composition (fat mass, lean mass and water) was determined at 7 months in awake mice by using nuclear magnetic resonance technology with an EchoMRI-700™ instrument (EchoMedical Systems, Houston, TX, USA). The mice were killed one week later. Data were analyzed using the manufacturer’s software: Windows XP Professional Edition, Microsoft.

### Echocardiography and Blood Pressure Measurement

Transthoracic echocardiography was consecutively performed in 3 to 7-month-old mice (separate set of experiments) and in 7-month-old mice two days before sacrifice, using the Vevo770™ imaging system (VisualSonics Inc., Toronto, Canada), equipped with a 30 MHz probe. Briefly, the mice were anesthetized with isoflurane and their body temperature was maintained at 37°C with an infrared lamp. The heart was imaged in the long-axis and short-axis view of the left ventricle (LV), with 3 to 5 measurements for each view in order to make an average. The cursor of the M-mode was positioned perpendicular to the anterior wall in order to measure left ventricular end-diastolic and end-systolic diameters (LVEDD and LVESD, respectively) at the level of papillary muscles below the mitral valve tip. Heart rate, fractional shortening (FS) and left ventricular ejection fraction (LVEF) were calculated.

Systolic blood pressure (SBP) and diastolic blood pressure (DBP) were measured using a validated tail-cuff method in anesthetized mice just before the echocardiography. For each mouse, the average of 3 measurements for both SBP and DBP was calculated.

### Plasma and Tissue Sampling

At either 24 days or 7 months old, and after 12-hours fasting, mice from the NF and OF groups were profoundly anesthetized with sodium thiopental (60 mg/kg, i.p.) until total loss of nociceptive reflexes (verified by paw pinching), and heparin was injected (500 UI/kg, i.p.). Blood was collected from the abdominal aorta, and immediately centrifuged at 4°C in order to separate the plasma, and samples were stored at −80°C until subsequent measurements. The hearts were quickly harvested in order to determine gene expression (24 days), oxidative stress markers (7 months old), collagen deposition (7 months old), or to determine ex-vivo susceptibility to ischemia reperfusion injury (7 months old, separate groups of mice). After harvesting, the hearts for gene expression and ventricular antioxidant activity were frozen in liquid nitrogen and stored at −80°C until subsequent measurements.

### Microarray Analysis

Gene expression profiles were determined in the hearts of NF and OF mice at 24-days of age.

### Extraction of Total RNA

Total RNA was extracted from heart samples by Quiazol reagent (Invitrogen, Life Technologies, Saint Aubin, France). The quality and concentration were checked using Agilent technology. Four samples from each age (4 NF and 4 OF, from 24-day-old mice) with sufficient yield and integrity for complete microarray analysis were isolated. Good-quality RNA was amplified and antisense RNA were coupled with Cy3 using the Low RNA input QuickAmp kit (Agilent Technologies), according to the manufacturer’s instructions.

### Hybridization on Microarrays

Transcript profiling was performed using mouse Agilent 8*60 k arrays (G4852A). These microarrays contained 39,430 distinct oligonucleotide probes (60 mers) and 16,251 lincRNAs. The list of probes is available online (http://www.chem.agilent.com). Microarray hybridization with labeled-RNA was carried out by the functional genomic platform of the Institut de Pharmacologie Moléculaire et Cellulaire (CNRS, Sophia-Antipolis, France). Quantile normalization was used to level out the magnitude of the measurements. The statistical analysis was done using Limma (Linear Models for Microarray Data). The microarray results were deposited in the GEO database (http://www.ncbi.nlm.nih.gov/geo/) under GEO Series Accession Number GSE38995.

### Real-time Quantitative PCR for Specific Gene Expression

mRNA (1 µg) was reverse transcribed with MMLV reverse transcriptase (Invitrogen, id.) using random hexamers (Invitrogen, id.) according to the manufacturer’s instructions. Real-time quantitative polymerase chain reaction (RT-PCR) was performed with 2 µl of cDNA using the SYBR-Green PCR Master-Mix (Applied Biosystems) and both sense and antisense primers (5 µM) in a final volume of 20 µL, in a 7500-Fast-Real-Time PCR system (Applied Biosystems). The primers used for the amplification of mouse genes are provided in Table 1. Target gene expression was normalized against the gene expression of housekeeping hypoxanthine-guanine phosphoribosyltransferase (HPRT).

**Table 1:** Forward and reverse sequences of primers used for the amplification of mouse genes.

Gene	Forward (5′ to 3′)	Reverse (3′ to 5′)
HPRT	CTGGTGAAAAGGACCTCTCG	TGAAGTACTCATTATAGTCAAGGGCA
Apelin	CCTTGACTGCAGTTTGTGGA	CTCGAAGTTCTGGGCTTCAC
Apelin receptor (APJ)	ACCTTTGTGGTGACTTTGCC	GCAAAAGACACTGGCGTACA
MnSOD	AACTCAGGTCGCTCTTCAGC	GCTTGATAGCCTCCAGCAAC
Catalase	CCGCAATCCTACACCATGTC	TATCTCCTATTGGGTTCCCG
MMP-2	GAGAAAAGCGCAGCGGAGTGACG	TTCCCCCGCAAGCCCAAGTG

HPRT: hypoxanthine-guanine phosphoribosyltransferase; MnSOD: manganese-superoxide dismutase; MMP-2: matrix metalloproteinase type-2.

### Biochemical Measurements

Plasma glucose concentrations were determined using a glucometer, and plasma insulin, leptin, adiponectin and apelin were determined by EIA using specific commercial kits (Gentaur, Paris, France except for apelin Euromedex, Souffelweyersheim, France). Cholesterol was assayed on a Victor 1420 Multilabel Counter (Wallac-Perkin Elmer Life Sciences, Courtaboeuf, France) using a commercially available kit (Diasys, Condom, France).

### Oxidative Stress Measurements

#### Plasma oxidative stress level

Oxidative stress was determined in 10 µL of plasma by the free oxygen radical test (F.O.R.T, FORM-PLUS-3000, Optimabio, Ollioules, France). The color intensity correlates directly with the amount of hydroperoxide and, consequently, with the level of oxidative stress [6].

### Measurement of Reactive Nitrogen-oxygen Species in the Heart Using Electron Spin Resonance (ESR) Spectroscopy

Freshly harvested hearts were perfused with ice-cold Krebs-Henselheit buffer to remove residual blood. Myocardial biopsies of 3 mm diameter were rinsed, weighed and placed in a 24-well tissue culture plate containing 200 µL of ESR-specific pH 7.4 Krebs-HEPES buffer, to which deferroxamine (25 µM) and diethyl-dithiocarbamate (5 µM) were added to remove traces of contaminant metal ions. The spin probe 1-hydroxy-3-methoxy-carbonyl-2,2,5,5- tetramethyl pyrrolidine (CMH) was added in order to reach a final concentration of 100 µM, then the tissue was incubated for 15 minutes at 37°C, in a 5% CO_2_ atmosphere, in the presence of CMH. In the presence of reactive nitrogen-oxygen species, the ESR-silent CMH hydroxylamine is oxidized into CP^•^ nitroxide radical. The buffer and tissue were collected and immediately frozen in liquid nitrogen then analyzed at 100K in a Bruker X-band EMX spectrometer (Wissembourg, France) using an HS cavity. The height of the central anisotropic peak of the CP^•^ signal was measured (in arbitrary units, AU) and the results were expressed in AU/mg of fresh tissue.

### Antioxidant Enzyme Activity in the Heart

Superoxide dismutase (SOD) was measured in heart samples using a method described previously [7]. Catalase activity in the heart was measured using a modified method [8] derived from Aebi [9]. All cardiac enzyme activities were related to protein content determined by the Lowry method.

### Ventricular Remodeling

#### Collagen content

Intact ventricles from freshly-harvested hearts were fixed in 4% paraformaldehyde. Histological analysis was used to determine the development of cardiac fibrosis by staining paraffin sections for total collagen with picrosirius red as described before [4].

#### Matrix metallo-proteinase-2 (MMP-2)-associated gelatinase activity

Proteins were extracted from frozen intact heart tissue in lysis buffer containing 250 mM Tris-HCl (pH 7.4), 200 mM NaCl, Nonidet-P40 1%. Matrix metallo-proteinase-2 (MMP-2)-associated gelatinase activity was determined as described before [4].

### Susceptibility to ex vivo Ischemia/Reperfusion (I/R) Injury

#### Preparation of isolated hearts

After the mice had been anesthetized with sodium thiopental (60 mg/kg, i.p.), heparin was injected (see previously) and the hearts were excised and placed in a cold (4°C) perfusion buffer bath until contractions ceased. The hearts were cannulated in the Langendorff mode, as described previously [4]. Coronary flow, heart rate (HR) and left ventricular pressures: left ventricular end-diastolic pressure (LVEDP), left ventricular end-systolic pressure (LVESP), left ventricular developed pressure (LVDP = LVESP-LVEDP), and the first derivative of LVDP, left ventricular maximal pressure development (+dP/dt) and relaxation (−dP/dt) were recorded throughout the experiments.

#### Ischemia/Reperfusion (I/R) protocol

The isolated hearts were initially perfused in the Langendorff mode for 20 minutes for stabilization. The cardiac parameters were then recorded for 12 min, after which the perfusion was turned off in order to induce 30 min of total global normothermic ischemia, which was followed by reperfusion in the Langendorff mode for 2 hours.

#### Infarct size

At the end of 2 h of reperfusion, the hearts were cut into five transverse slices, each about 1 mm thick. These slices were then incubated in triphenyl tetrazolium chloride (TTC) solution in phosphate buffer (pH 7.4, 37°C) for 20 minutes. After incubation in TTC, the slices were photographed from both sides and contoured with image analysis software (Histolab) to delineate the borders of the entire heart and the infarcted area (white). The infarct size was calculated as a percentage of the total myocardial area.

#### Western blot analysis

Phosphorylated states of signal transducer and activator of transcription-3 (phospho-STAT-3 Tyr 705) and total levels of STAT-3 were analyzed by sodium dodecyl sulfate-polyacrylamide gel electrophoresis with antibodies (Cell Signaling, Ozyme, Saint Quentin Yvelines, France) as described elsewhere [10], in protein lysates extracted from intact heart ventricles from 7-month-old mice from both groups. The expression of suppressor of cytokine signaling-3 (SOCS-3) was also analyzed by the same method with antibodies (Cell Signaling, Ozyme, Saint Quentin Yvelines, France), and normalized to the expression of HSC70. Equal loading was verified with Ponceau staining and levels of phosphorylated proteins were normalized to their total protein levels performed in the same samples and under the same conditions but on a separate membrane. Relative densitometry was determined using computer software.

### Statistical Analysis

The results are expressed as mean values ± SEM. The statistical significance of differences between two means was determined by a Student’s *t*-test. For weight gain, repeat ANOVA was performed to investigate differences between the two groups.

## Results

### Body Weight and Body Composition

**Figure 1:**
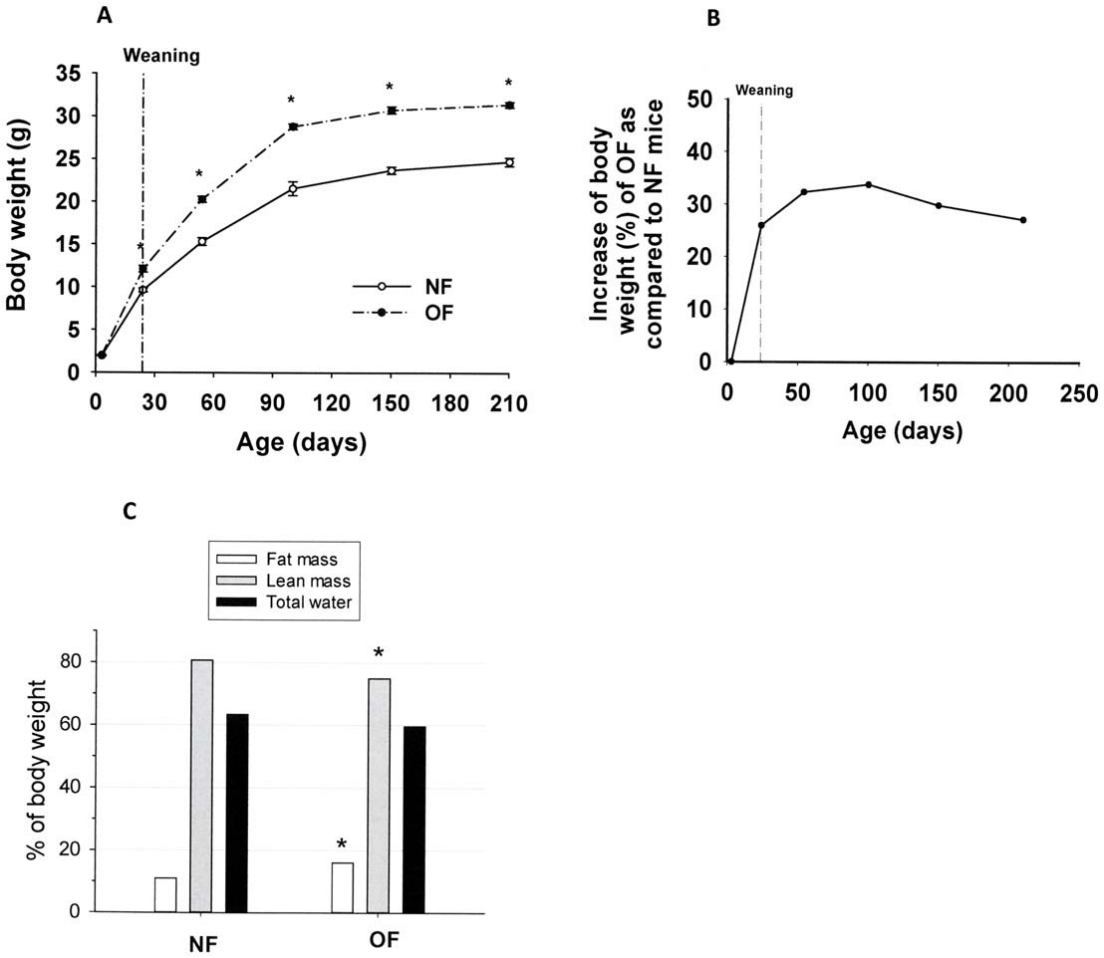
Increase in body weight and body composition during life in mice postnatally overfed (OF) by litter size reduction (3 pups/litter) or normally fed (NF) in litters of normal size (10 pups/litter). A: Body weight from birth to 210 days (7 months) of OF and NFmice. Results are expressed as means ± SEM. *, P<0.05 was considered significant (one-way ANOVA). B: Percentage of mean body weight increase in OF mice as compared with that in NF mice. C: Body composition (fat mass, lean mass and total water), measured by nuclear magnetic resonance, as a percentage of body weight in OF mice and NF mice. *, P<0.05 was considered significantly different. Data are expressed as means ± SEM from 10 animals per group.

Weight gain was significantly greater in overfed pups during the suckling period (figure 1A). At weaning (day 24), the body weight of OF mice was 30% greater than that of NF mice. This difference persisted in mature mice (figure 1B). This higher body weight was associated with a significantly greater body fat mass (+47%) and lower lean mass (−7%) in OF mice (figure 1C).

### Biochemical Parameters

As shown in Table 2, total cholesterol in 7-month-old OF mice was 15% greater than that in NF mice. Plasma levels of fasting insulin and leptin were also significantly greater in OF mice than in NF mice (p<0.05). Fasting plasma glucose, adiponectin and apelin in the two groups were no different.

**Table 2:** Metabolic parameters of NF and OF mice groups. Fasting blood glucose was assayed by glucometry; cholesterol and triglycerides were assayed using colorimetric methods and plasma insulin, leptin and adiponectin were estimated using ELISA. P was calculated by Student’s *t*-test.

		NF		OF
Fasting glucose (mmol/l)		10.27±0.61		10.36±0.59
Cholesterol (g/l)		0.68±0.03		0.78±0.02*
Insulin (ng/ml)		0.99±0.08		1.64±0.19*
Leptin (ng/dl)		0.39±0.02		0.91±0.16*
Adiponectin		16.56±2.35		9.02±0.92
Apelin (pg/ml)		3.31±0.03		3.32±0.04

* , *P*<0.05, NF vs. OF group.

### Microarrays Analysis

RNA samples of 24-day-old hearts were analyzed. In these hearts, we isolated 822 genes that were differentially expressed in OF and NF mice (with p<0.05). Using log2 signal<7+?log2ratio?>0.7+lodScore>2 as a new filter, we restricted our results to 102 targets corresponding to 35 under-expressed and 67 over-expressed genes; the full list of these 102 genes classified according to the seven functional categories described by Hwang et al. [11], is presented in Data Supplement Table 1. Among these 102 differentially expressed genes, structural genes accounted for 12% of over-expressed genes, principally collagen encoding genes (Table 3), in OF mice. Moreover, we noted that the apelin receptor (APJ) gene was strongly over-expressed (ratio: 1.80, p = 0.012) and its intracellular negative regulator, Rab7, was under-expressed in OF mice (ratio = 0.80, p = 0.032).

**Table 3:** List of genes differentially expressed between NF and OF mice at 24 days of age.

Gene	Description		FD	p
*Structure genes*				
**Ctnna3**		**catenin (cadherin associated protein), alpha-3**	**0.70**	**0.013**
**Dmd**		**dystrophin, muscular dystrophy**	**0.66**	**0.022**
**Mylk3**		**myosin light-chain kinase-3**	**0.65**	**0.023**
**Acta1**		**actin, alpha-1, skeletal muscle**	**2.03**	**0.012**
**Col14a1**		**collagen, type XIV, alpha-1**	**1.50**	**0.024**
**Col15a1**		**collagen, type XV, alpha-1**	**1.44**	**0.019**
**Col20a1**		**collagen, type XX, alpha-1**	**1.50**	**0.022**
**Col5a1**		**collagen, type V, alpha-1**	**1.53**	**0.018**
**Col6a1**		**collagen, type VI, alpha-1**	**1.41**	**0.024**
**Emilin1**		**elastin microfibril interface-1**	**1.37**	**0.024**
**Myl1**		**myosin, light polypeptide-1**	**1.89**	**0.017**
Actn1		actinin, alpha-1	1.29	0.040
Col16a1		collagen, type XVI, alpha-1	1.35	0.048
Col1a1		collagen, type I, alpha-1	1.50	0.036
Col3a1		collagen, type III, alpha-1	1.67	0.033
Col4a4		collagen, type IV, alpha-4	1.38	0.034
Col6a2		collagen, type VI, alpha-2	1.36	0.045
Col6a3		collagen, type VI, alpha-3	1.40	0.036
Dync2li1		dynein cytoplasmic-2 light-intermediatechain-1	1.36	0.045
Fbln7		fibulin-7	1.43	0.027
Sept9		septin-9	1.35	0.035
Vim		vimentin	1.34	0.040
*Other genes*				
**Ednrb**		**endothelin receptor type-B**	**0.63**	**0.012**
**Edn1**		**Endothelin-1**	**1.39**	**0.019**
**Aplnr**		**apelin receptor**	**1.80**	**0.012**
**Gsta1**		**glutathione S-transferase, alpha-1**	**1.50**	**0.013**
**Igf1**		**insulin-like growth factor-1**	**1.39**	**0.024**
**Igfbp4**		**insulin-like growth factor binding protein-4**	**1.48**	**0.017**

Bold genes are genes extracted from the list of 102 genes and the others are from the list of 822 genes.

**Figure 2:**
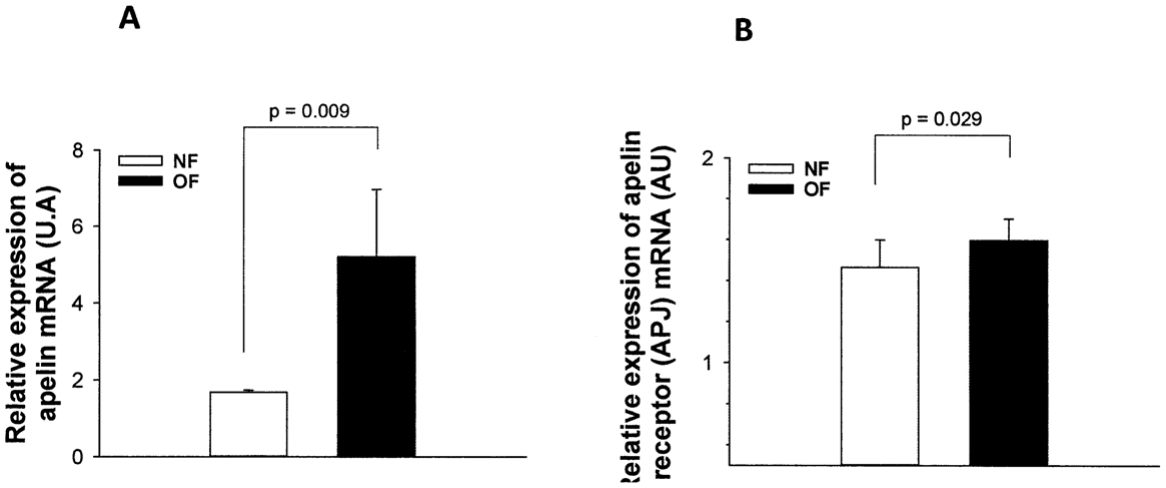
Apelin and apelin receptor (APJ) gene expression in myocardial tissue mRNA extracts from young (24 days) postnatally normal-fed (NF) or overfed (OF) mice. The mRNA expression of (A) apelin and (B) apelin receptor genes was measured by quantitative RT-PCR in myocardial tissues extracted from juvenile (24 days) mice that were overfed (OF) by litter size reduction (3 pups/litter) or normally fed (NF) in litters of normal size (10 pups/litter). Results are expressed as means ± SEM from 8 animals per group. P was calculated by Student’s *t*-test.

Quantitative real-time PCR analysis performed on 24-day-old mouse heart mRNA extracts confirmed the relative over-expression of APJ, but also of apelin itself in young postnatally overfed mice (figure 2 A and B). The relative expressions of these genes were not different in 7-month-old OF and NF mice (data not shown).

### Oxidative Stress Measurements in Plasma and Tissues

**Figure 3:**
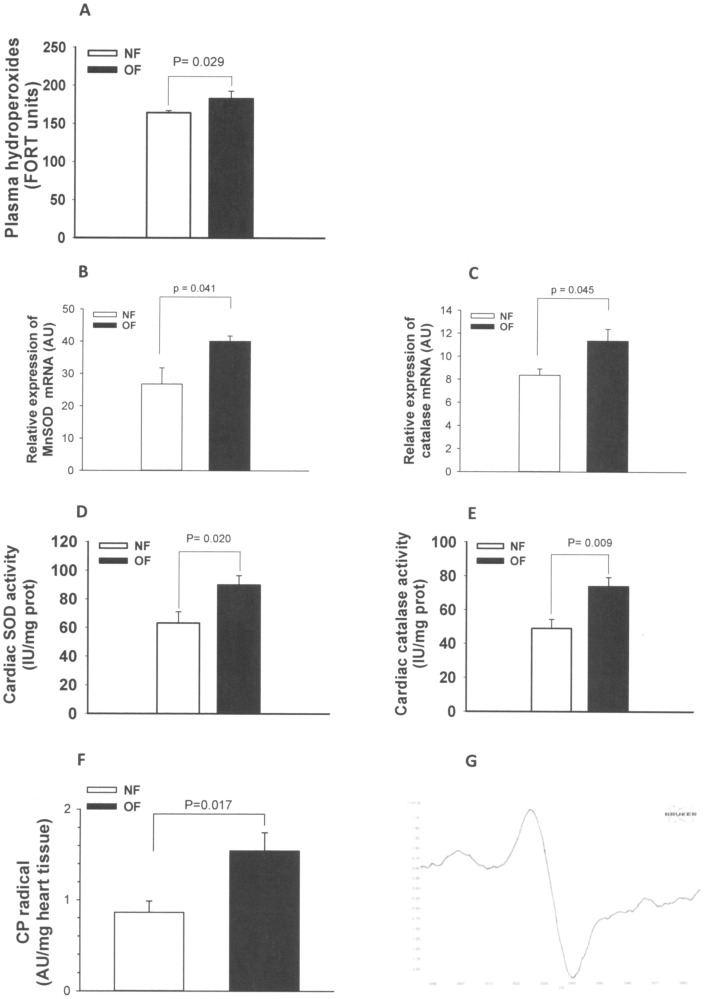
Oxidative stress indexes in plasma and heart tissue of 7-month-old mice that were postnatally overfed (OF) by litter size reduction (3 pups/litter) or normally fed (NF) in litters of normal size (10 pups/litter). A: Plasma hydroperoxides, considered as a marker of peroxidation in the circulation, were measured in OF and NF mice (FORT test). B: Cardiac MnSOD mRNA relative expression measured by quantitative RT-PCR in myocardial tissues extracted from adult (7 months) NF or OF mice. C: Cardiac catalase mRNA relative expression measured by quantitative RT-PCR in myocardial tissues extracted from adult (7 months) NF or OF mice. D: Cardiac global superoxide dismutase (SOD) enzymatic activity. E: Cardiac catalase enzymatic activity. F: CP^•^ nitroxide radical formation in heart tissue, measured with electron paramagnetic resonance spectroscopy. Under the presence of reactive nitrogen and oxygen species, the cell-permeable CMH hydroxylamine probe is converted into the nitroxide CP^•^ radical, which can be considered a marker of tissue nitro-oxidative stress. G: Representative anisotropic signal of CP^•^ radical in heart tissue at 100K. Data are expressed as means ± SEM from 10 animals per group. P was calculated by Student’s *t*-test.

We observed significantly higher plasma levels of hydroperoxides (figure 3A) in the OF group than in the NF group.

In heart ventricular tissue, a characteristic anisotropic signal corresponding to the oxidation of CMH into CP^•^ nitroxide radical by reactive nitrogen-oxygen species was observed (figure 3C). After quantification of the signals, we found a far higher level of CMH oxidation (+79%) in myocardial biopsies from OF mice (figure 3B).

In heart ventricular tissue from 7-months old mice, expression of Mn-SOD and catalase mRNA (figure 3D and 3E) as well as global SOD and catalase activity were significantly higher in the OF group than in the NF group (figure 3F and 3G). No differences were found in juvenile hearts from 24-days-old OF and NF mice for the gene expression of these antioxidant enzymes (data not shown).

### In vivo Cardiac and Vascular Function

**Figure 4:**
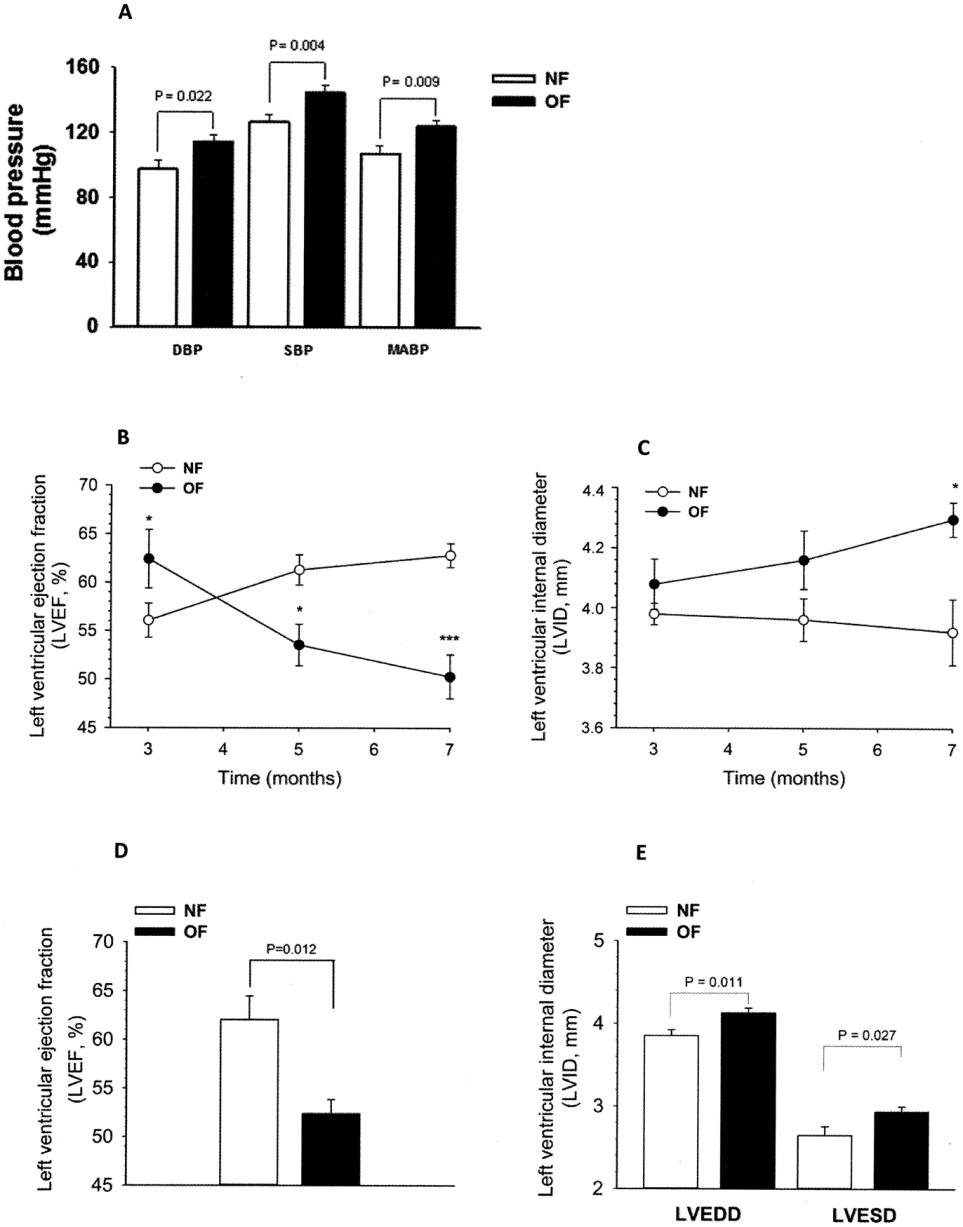
Cardiovascular function in anesthetized 7-month-old mice that were postnatally overfed (OF) by litter size reduction (3 pups/litter) or normally fed (NF) in litters of normal size (10 pups/litter). A: Diastolic (DBP), systolic (SBP) and mean arterial blood pressure (MABP) measured at 7 months by plethysmography. B: Evolution of left ventricular ejection fraction (LVEF, in %) from 3 months to 7 months of life, measured in vivo by echocardiography. C: Evolution of left ventricular internal diameter (LVID) from 3 months to 7 months of life, measured in vivo by echocardiography. *, P<0.05; ***, P<0.001 were considered significantly different from NF mice. D: left ventricular ejection fraction measured at 7 months in anesthetized NF and OF mice by echocardiography (LVEF, in %). E: left ventricular end diastolic diameter (LVEDD) and left ventricular end systolic diameter (LVESD) measured at 7 months in anesthetized NF and OF mice by echocardiography. Data are expressed as means ± SEM from 10 animals per group. P was calculated by Student’s *t*-test.

In 7-month-old anesthetized mice diastolic, systolic and mean arterial blood pressure was slightly but significantly higher (+10 mmHg) in the OF group (figure 4A). There was no significant difference between the groups for heart rate.

Interestingly, the ejection fraction, the proportion of left ventricular end-diastolic volume that is efficiently ejected through the aorta during the systole, that was initially slightly greater in 3-month-old OF mice, gradually decreased over time and was significantly lower in OF than in NF mouse hearts at 5 and 7 months (figure 4B and D). Additionally, left ventricular end-diastolic and end-systolic diameters increased gradually with age and were both greater in 7-month-old OF mice (figure 4C and E). The weight of OF hearts, measured ex-vivo during sacrifice, was slightly greater, but the heart to body weight ratio, a measurement of heart hypertrophy, was similar in OF and NF mice.

### Ventricular Remodeling

**Figure 5:**
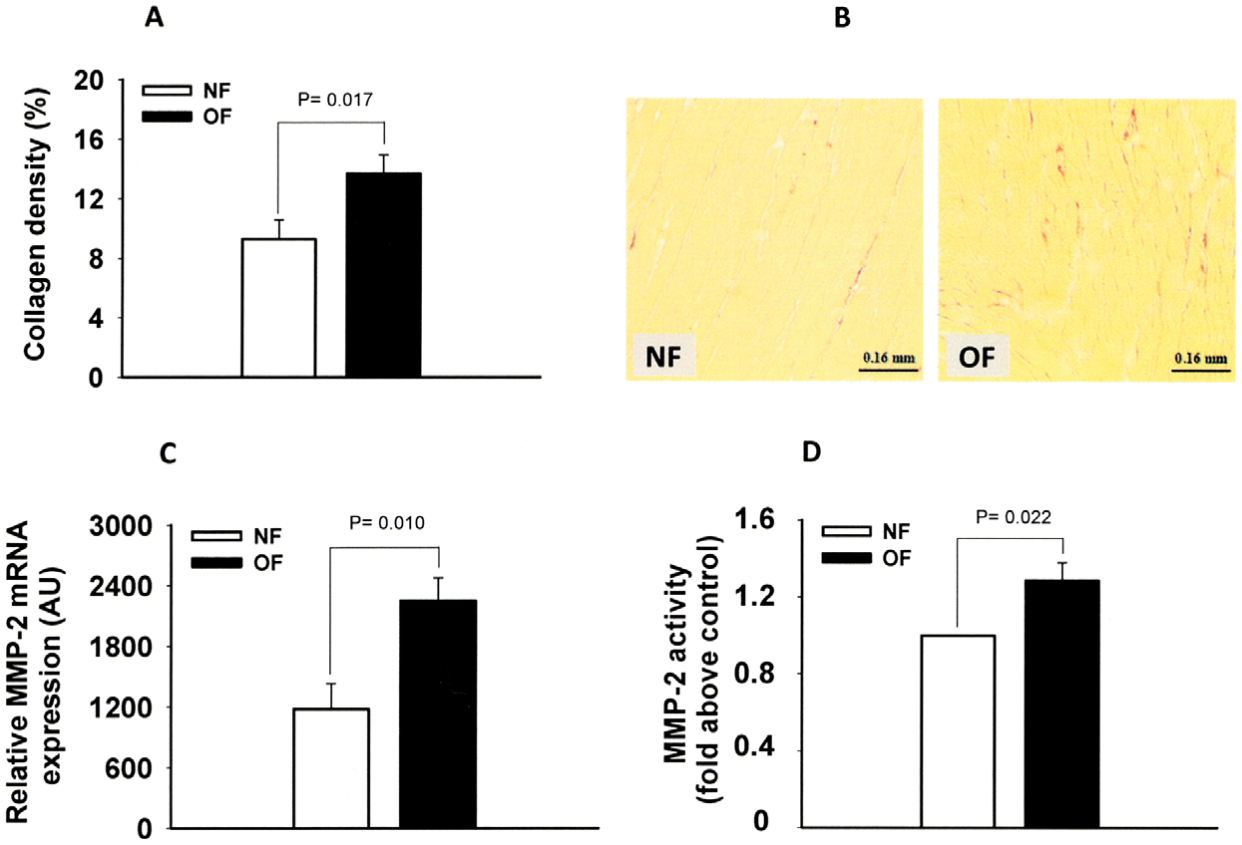
Ventricular remodeling of hearts from 7-month-old NF or OF mice. A: Collagen density (% of area) of ventricular tissues from 7 months-old NF and OF mice. B: Representative picrosirius collagen staining of ventricular tissue from 7 months-old NF and OF mice., C: MMP-2 mRNA expression in myocardial tissues from 7 months-old NF and OF mice, measured by quantitative RT-PCR, D: Metalloproteinase activity in myocardial tissues from 7 months-old NF and OF mice, measured by gelatin zymography. P was calculated by Student’s *t*-test.

In intact hearts, histological studies showed a higher collagen density in adult OF mice ventricles (Figure 5A and 5B). The analysis of MMP-2 expression and activity showed greater MMP-2 expression (figure 5C) and activity (figure 5D) in OF mice heart ventricular tissue.

### Heart’s Susceptibility to Ischemia/Reperfusion Injury

**Figure 6:**
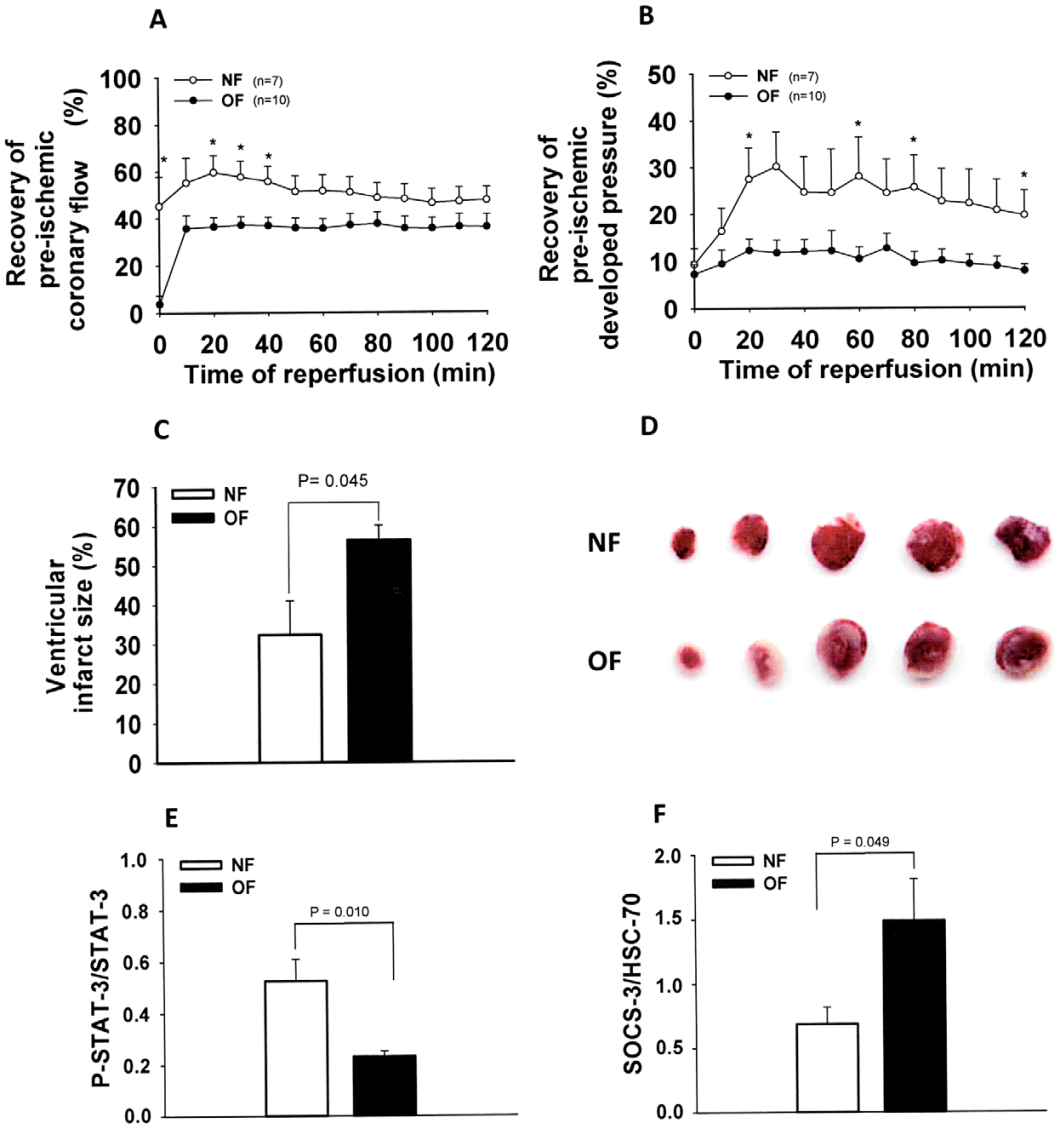
Myocardial contractility recovery, tissue injury of hearts from 7-month-old NF or OF mice, after 30 min of global normothermic ischemia followed by 2 hours of reperfusion, and expression of STAT-3 and SOCS3 in intact (non-ischemic) ventricles of 7-months old NF or OF mice. A: Recovery of pre-ischemic coronary flow (in %) of isolated Langendorff perfused hearts after 30 min of global normothermic total ischemia. B: Recovery of pre-ischemic developed pressure (in %) of isolated Langendorff perfused hearts after 30 min of global normothermic total ischemia. C: Ratios of ventricular infarcted areas/non-ischemic tissue (in %) of NF and OF mice hearts, after 30 min of global ischemia followed by 2 h of reperfusion. P was calculated by Student’s *t*-test. D: representative pictures of TTC-staining of myocardial slices of NF and OF mice after ischemia-reperfusion. Red-stained areas show non-ischemic tissue and white areas show infarcted tissue. E: Phosphorylated/total STAT-3 protein expression in intact ventricular tissue (non-ischemic) of NF and OF mice. F: SOCS-3/HSC70 protein expression measured by Western blot in intact ventricular tissue (non-ischemic) of NF and OF mice.

In baseline perfusion conditions, there was no difference between OF and NF mice in all of the cardiac parameters measured. After 30 minutes of ischemia, reperfusing the myocardium led to progressive but incomplete recovery of the heart’s circulating and contractile activity. In OF mouse hearts, the recovery of coronary flow (figure 6A) of LVDP (figure 6B), +dP/dt and –dP/dt were significantly impaired in comparison with these of NF mice. In addition, the evaluation of still viable (red) and necrotic zones (white) after 2 hours of reperfusion showed a far greater infarcted area (+74%) in OF hearts (figures 6C and 6D).

Western blot analysis of intact ventricular heart tissue revealed a decrease in tyrosine phosphorylated/total STAT-3 in hearts of OF mice (Figure 6E, p<0.01) and an increase in the expression of SOCS-3 (Figure 6F, p<0.05).

## Discussion

Our results show for the first time that the nutritional environment during the postnatal period induces early changes in heart gene expression that may have a long-term effect on cardiovascular function and heart structure in adult mice; and may have a deleterious impact on myocardial injury recovery after an ischemic insult.

Our study provides new original data and bring to light the fact that nutritional conditions in immediate postnatal life, such as overnutrition, may influence the expression of several genes involved in the heart’s structural organization (collagen, myosin, actin, dystrophin, dynein …) metabolism and somatotropic system (insulin-growth factors), vasoreactivity (endothelin-1 and receptor), ion channels (Scn4b), cell signaling/communication (APJ) and oxidative stress (glutathione S-transferase). It is noteworthy that these differences occurred in pups that were randomly assigned to either small or large litters, and might not be related to genetic differences due to distinct ascendants. Therefore, after only 24 days of increased milk consumption, myocardial genome expression was modified in overfed mice and may have induced some permanent changes in the cytoskeleton and organization of cardiomyocytes, even if microarrays were processed in whole heart ventricles, which contain several types of cells such as endothelial, vascular smooth muscle and fibroblastic cells in addition to cardiomyocytes. Thus, additional data are now necessary to confirm the changes detected by microarrays at the mRNA and protein levels, but also to determine which cell types are concerned by these modifications.

In the present study, we found that postnatal over-feeding (OF) induced a significant increase in body weight at weaning (+30%) which persisted at the same level with maturation. Interestingly, this increase in body weight was found to be related to an elevation of body fat mass, at the expense of lean mass. Experimental evidence during excision of the hearts revealed that adipose tissue deposition was mainly visceral but not subcutaneous, which is in favor of the “central adiposity” concept. These observations concerning increased body weight are consistent with an earlier study [5], with some differences probably due to the number of pups per mother, the strains and the composition of the standard diet.

Postnatally overfed mice displayed alterations in various biochemical parameters, such as increased plasma insulin and cholesterol. Our results confirm a previous study performed in 12-month-old mice [5] which showed increased HDL-cholesterol, triglycerides and free fatty acids. In this previous study as in ours, no modifications in fasting glucose were observed, despite alterations in glucose tolerance and a major increase in plasma insulin at weaning and later. In fact, hyperinsulinemia starts in the earliest stages of life in OF pups, and leads to permanent insulin resistance at both the hypothalamic [12] and peripheral [13] levels.

Given that OF mice had increased adipose mass, we also assessed the plasma levels of some adipokines, such as leptin, adiponectin and apelin and found higher levels of leptin in OF mice but no differences for levels of adiponectin and apelin. Leptin plays a major role in the regulation of appetite and energy balance, but it also induces a variety of actions in the cardiovascular system. An increase in leptin levels in OF rats [3], [14]–[16] or mice [5] is consistently reported in the literature, and can be explained by the fact that leptin levels are related to the amount of fat mass. Though OF mice have increased circulating levels of leptin, previous studies in rats suggested that overfeeding may induce central leptin resistance, due to lower Ob-Rb expression in the hypothalamic nucleus [16], [17].

Concerning adiponectin, the results are less consensual, but previous studies performed in rats showed no difference between OF and NF groups at 4 and 6 months [3]. Adiponectin production is inversely proportional to whole body adipose mass, and experimental studies suggest that it modulates the action of insulin and thus improves insulin sensitivity in peripheral tissues and induces general cardioprotective effects [18].

Microarray results obtained in juvenile hearts, which showed higher expression of apelin receptor in OF mice, encouraged us to explore some of the components of this adipocytokine system more thoroughly, given its increasing pathophysiological significance in nutritional disorders [19]. We report here that, despite no changes in circulating levels of apelin in young 24-day-old (data not shown) or adult postnatally overfed mice, local changes in this adipocytokine and its receptor may occur in the myocardium of young mice that were overnourished in the immediate postnatal period. In the cardiovascular system, apelin and APJ are abundantly expressed in the heart [20], [21]_ENREF_24. Apelin increases endothelium-dependent vasodilation [22] and was found to exhibit powerful inotropic activity [23]. Additionally, apelin may play an important role in cardiomyocyte specification and heart development [24]. Therefore, we speculate here that a modification in apelin receptor signaling in early postnatal life may permanently affect the development of the myocardium, a situation that may ultimately impair myocardial function.

Obesity and overweight are associated with increased oxidative stress not only in the bloodstream, but also in myocardial tissue [25]. In a previous study performed in OF rats in our group [4], we observed higher levels of plasma hydroperoxides and lower levels of vitamin C indicating exacerbated circulating oxidative stress, and the present work in OF mice confirms these initial data. Moreover, postnatal over-nutrition in mice also induced an increase in oxidative stress levels in the adult myocardial tissue, as evidenced by our ESR spectroscopy investigations. ESR is one of the few techniques that allow the direct measurement of free radical species; however, most biological reactive oxygen and nitrogen species (RONS) of interest are too short-lived to be measured directly with ESR spectroscopy. Hydroxylamine spin-probes such as CMH can be oxidized by superoxide anion or peroxynitrite, giving rise to a more stable long-lived ESR-detectable CP^•^ nitroxide, allowing detection of these unstable species in cell cultures or isolated organs [26]. This observation of increased nitro-oxidative stress in myocardial tissue of postnatally OF mice is a very original finding, and confirms the association between obesity and increased myocardial oxidative stress [27]. We also observed a significant increase in antioxidant SOD and CAT activities in heart tissue homogenates. At first sight, this appears to be a paradoxical result, but may reflect the adaptive response to increased oxidative stress.

Since postnatally overfed mice displayed metabolic alterations and increased oxidative stress, we investigated the consequences on cardiovascular parameters. First, early postnatal over-nutrition led to modifications in basal cardiovascular parameters in anesthetized mice. These included a discrete elevation in arterial blood pressure, enlarged left ventricles, and impaired left ventricular ejection fraction. To date, none of these modifications have been reported in overweight mice. Secondly, we explored the possibility of ventricular remodeling and found both greater collagen content and greater MMP-2 expression and activity in the ventricles of 7-month-old OF mice, showing an alteration in the architectural organization in the hearts of these mice, reinforcing our previous data obtained in rats [4]. These changes in myocardial organization in hearts might be the late consequences of the early modifications observed in gene expression of some isoforms of actin, myosin, collagen, dystrophin and other structural proteins that may have had a permanent impact on intracellular and organ structure. Indeed, the change in ventricular compliance could explain the impairment of ventricular ejection fraction and the enlargement of ventricular diameter. Several environmental factors such as oxidative stress or increased leptin levels might also explain the occurrence of this remodeling phenomenon. Indeed, studies have highlighted the role of leptin and insulin in the elongation of cardiac myocytes [28], ventricular hypertrophy [29], and heart failure [30].

Thirdly, we examined the recovery of pre-ischemic coronary flow, ventricular developed pressure, contractility and relaxation after I/R injury performed in vitro in isolated hearts, without any potential influence of neuro-humoral regulatory pathways. Langendorff hearts showed no differences in baseline measurements whereas the in vivo hearts did. Indeed, the conditions observed in vivo in anesthetized mice and in vitro in isolated perfused hearts differed considerably. Langendorff perfused heart must be considered a very simple tissue perfusing system, but is an essential model to explore cardiac function without any influence of endocrine or neuronal regulation, and it is very helpful to investigate myocardial response to global ischemia-reperfusion injury. We found that postnatal OF induced higher susceptibility of the heart to ischemia-induced injury, as shown by lower recovery of all of the pre-ischemic cardiac parameters and a markedly greater area of infarct. Therefore, in a stressful condition such as I/R injury, which generates a massive release of reactive oxygen-nitrogen species [31], the metabolic alterations induced by OF renders the myocardium more susceptible to damage. Additionally, the increase in collagen content and in MMP observed in ventricular tissue could also be a mediator for increased susceptibility to myocardial ischemia-reperfusion injury.

In intact (non-ischemic) ventricles, we found that the phosphorylation of STAT-3 was decreased and that the expression of SOCS-3 was increased in OF mouse hearts. STAT-3 is one of the seven isoforms found in the heart, and is a part of the “Survivor activating factor enhancement” (SAFE) pathway, which behaves as a critical molecule that protects against I/R injury [32], [33]. Indeed, STAT-3 is activated by ischemic pre- and post-conditioning [34] and various pharmacological cardioprotective agents [35]. SOCS-3 is a member of the STAT-induced STAT inhibitor, and acts as a suppressor of cytokine signaling (SOCS), inhibiting STAT-3 phosphorylation. Therefore, SOCS-3 may play a pivotal role in mediating both myocardial sensitivity to I/R injury and leptin resistance, as was recently described in the kidney of postnatally OF rats [36]. We therefore suggest that postnatal overnutrition may induce an increase in SOCS-3 expression, probably related to peripheral leptin resistance, which in turn inhibits the phosphorylation of STAT-3, thus impairing the SAFE cardioprotection pathway and rendering the hearts of OF mice more susceptible to I/R injury.

A cautionary note needs to be added concerning the use of genetically modified animals to evaluate the potential role of certain key protein genes and their impact on myocardial recovery after ischemia-reperfusion injury. Indeed, due to breeding difficulties, genetically modified mice are frequently raised in small litters, whereas their wild-type control mates are raised in normal (large) litters. According to our present data, caution should be done to compare genetically modified and wild-type mice originating from litters of equal numbers. Otherwise, some of the negative impacts of inactivating or up-regulating a gene in genetically modified mice could only be due to the long-term cardio-metabolic consequences of nutritional differences during the critical postnatal period.

To conclude, our study emphasizes that over-nutrition during the immediate postnatal period in mice leads to early changes in cardiac gene expression that may permanently modify the heart’s structural organization and metabolism and induce not only increases in myocardial markers of oxidative stress in adult mice, but also alterations in cardiovascular function and structure that are very likely to contribute to the greater susceptibility to myocardial injury after an ischemic insult. These results suggest that nutritional conditions in the immediate postnatal period should be taken into account since they may have an impact on cardiovascular risk in adulthood, and especially in the context of genetically modified mice that may be bred in small litters.

## Supporting Information

Table S1 **List of the 102 genes differentially expressed between NF and OF mice at 24 days of age.**
(DOC)
