# Supplementary material for: Postnatal Overfeeding Causes Early Shifts in Gene Expression in the Heart and Long-Term Alterations in Cardiometabolic and Oxidative Parameters
Source: PLoS One. 2013 Feb 26;8(2):e56981. doi: 10.1371/journal.pone.0056981 (PMC3582632; doi:10.1371/journal.pone.0056981)
Supplement: Table S1 — List of the 102 genes differentially expressed between NF and OF mice at 24 days of age. (DOC) [file pone.0056981.s001.doc]

| **Gene** | **Description** | **FD** | **p** |
| --- | --- | --- | --- |
|  |  |  |  |
| *Cell division* | |  |  |
| Ivns1abp | influenza virus NS1A binding protein | **0.42** | 0.005 |
| Ext1 | Exostosin | **0.63** | 0.017 |
| Rmnd5a | required for meiotic nuclear division 5 homolog A | **0.70** | 0.013 |
| Dap | death-associated protein | **1.41** | 0.018 |
| Mcm5 | minichromosome maintenance deficient 5 | **1.47** | 0.021 |
| Mcm2 | minichromosome maintenance deficient 2 mitotin | **1.50** | 0.017 |
| Casp8 | caspase 8 | **1.51** | 0.017 |
| Plk2 | polo-like kinase 2 | **1.55** | 0.018 |
| Ngfr | nerve growth factor receptor (TNFR superfamily, member 16) | **1.64** | 0.018 |
|  |  |  |  |
| *Cell signaling/communication* | |  |  |
| Slc5a3 | solute carrier family 5 (inositol transporters), member 3 | **0.61** | 0.013 |
| Ednrb | endothelin receptor type B | **0.63** | 0.012 |
| Cngb3 | cyclic nucleotide gated channel beta 3 | **0.63** | 0.006 |
| Rragd | Ras-related GTP binding D | **0.65** | 0.018 |
| Camk1g | calcium/calmodulin-dependent protein kinase I gamma | **0.68** | 0.024 |
| Art3 | ADP-ribosyltransferase 3 | **0.69** | 0.018 |
| Rrad | Ras-related associated with diabetes | **0.69** | 0.014 |
| Ctnna3 | catenin (cadherin associated protein), alpha 3 | **0.70** | 0.013 |
| Rilpl1 | Rab interacting lysosomal protein-like 1 | **0.71** | 0.022 |
| Plcd3 | phospholipase C, delta 3 | **0.71** | 0.013 |
| Ap2m1 | adaptor protein complex AP-2 | **0.72** | 0.025 |
| Lmo7 | LIM domain only 7 | **0.73** | 0.019 |
| Gab3 | growth factor receptor bound protein 2-associated protein 3 | **1.34** | 0.023 |
| Pdgfrb | platelet derived growth factor receptor, beta polypeptide | **1.38** | 0.020 |
| Edn1 | endothelin 1 | **1.39** | 0.019 |
| Igf1 | insulin-like growth factor 1 | **1.39** | 0.024 |
| Efemp2 | epidermal growth factor-containing fibulin-like extracellular matrix protein 2 | **1.39** | 0.022 |
| Megf6 | multiple EGF-like-domains 6 | **1.39** | 0.013 |
| Tmem106c | transmembrane protein 106C | **1.40** | 0.017 |
| Rcn3 | reticulocalbin 3, EF-hand calcium binding domain | **1.44** | 0.018 |
| Cacna1a | calcium channel, voltage-dependent, P/Q type, alpha 1A subunit | **1.44** | 0.015 |
| Figf | c-fos induced growth factor | **1.45** | 0.018 |
| Astn1 | astrotactin 1 | **1.46** | 0.024 |
| Arrdc4 | arrestin domain containing 4 | **1.47** | 0.017 |
| Olfml3 | olfactomedin-like 3 | **1.49** | 0.016 |
| Arl5c | ADP-ribosylation factor-like 5C | **1.49** | 0.020 |
| Slc16a11 | solute carrier family 16 (monocarboxylic acid transporters), member 11 | **1.51** | 0.013 |
| Lrrc17 | leucine rich repeat containing 17 | **1.55** | 0.017 |
| Tmem178 | transmembrane protein 178 | **1.56** | 0.024 |
| Lpar4 | lysophosphatidic acid receptor 4 | **1.61** | 0.024 |
| Mdk | midkine | **1.68** | 0.022 |
| Aplnr | apelin receptor | **1.80** | 0.012 |
| Scn4b | sodium channel, type IV, beta | **4.44** | 0.005 |
|  |  |  |  |
| *Cell structure/motility* | |  |  |
| Mylk3 | myosin light chain kinase 3 | **0.65** | 0.023 |
| Dmd | dystrophin, muscular dystrophy | **0.66** | 0.022 |
| Emilin1 | elastin microfibril interfacer 1 | **1.37** | 0.024 |
| Cplx2 | complexin 2 | **1.38** | 0.022 |
| Col6a1 | collagen, type VI, alpha 1 | **1.41** | 0.024 |
| Col15a1 | collagen, type XV, alpha 1 | **1.44** | 0.019 |
| S100a11 | S100 calcium binding protein A11 (calgizzarin) | **1.48** | 0.018 |
| Col20a1 | collagen, type XX, alpha 1 | **1.50** | 0.022 |
| Col14a1 | collagen, type XIV, alpha 1 | **1.50** | 0.024 |
| Col5a1 | collagen, type V, alpha 1 | **1.53** | 0.018 |
| Myl1 | myosin, light polypeptide 1 | **1.89** | 0.017 |
| Acta1 | actin, alpha 1, skeletal muscle | **2.03** | 0.012 |
|  |  |  |  |
| *Cell/organism defense* | |  |  |
| N4bp2 | NEDD4 binding protein 2 | **0.61** | 0.020 |
| Herc1 | hect (homologous to the E6-AP (UBE3A) carboxyl terminus) domain and RCC1 (CHC1)-like domain | **0.62** | 0.017 |
| Lrrc8a | leucine rich repeat containing 8A | **0.66** | 0.024 |
| Hmga1 | high mobility group AT-hook 1 | **0.74** | 0.018 |
| Gsta1 | glutathione S-transferase, alpha 1 | **1.50** | 0.013 |
| Thy1 | thymus cell antigen 1, theta | **1.53** | 0.024 |
| Ces2e | carboxylesterase 2E | **1.62** | 0.012 |
| Cstad | CSA-conditional, T cell activation-dependent protein | **2.28** | 0.005 |
|  |  |  |  |
| *Gene/protein expression* | |  |  |
| LOC790956 | 5.8S ribosomal RNA | **0.27** | 0.023 |
| Elk4 | ELK4, member of ETS oncogene family | **0.69** | 0.023 |
| Zbtb7a | zinc finger and BTB domain containing 7a | **0.71** | 0.017 |
| Tfdp2 | transcription factor Dp 2 | **0.75** | 0.020 |
| Hhex | hematopoietically expressed homeobox | **1.37** | 0.023 |
| Zfp608 | zinc finger protein 608 | **1.38** | 0.023 |
| Tcf3 | transcription factor 3 | **1.38** | 0.015 |
| Zfp423 | zinc finger protein 423 | **1.39** | 0.021 |
| Wdr6 | WD repeat domain 6 | **1.40** | 0.024 |
| Eme2 | essential meiotic endonuclease 1 homolog 2 | **1.44** | 0.024 |
| Rab37 | RAB37, member of RAS oncogene family | **1.47** | 0.018 |
| Nrip2 | nuclear receptor interacting protein 2 | **1.48** | 0.018 |
| Capn3 | calpain 3 | **1.50** | 0.023 |
| Tnfaip8l1 | tumor necrosis factor, alpha-induced protein 8-like 1 | **1.53** | 0.020 |
| Wdr86 | WD repeat domain 86 | **1.57** | 0.010 |
| Adamts17 | disintegrin-like and metallopeptidase (reprolysin type) with thrombospondin type 1 | **1.70** | 0.022 |
|  |  |  |  |
| *Metabolism* | |  |  |
| Atp6ap1l | ATPase, H+ transporting, lysosomal accessory protein 1-like | **0.38** | 0.005 |
| Gls | glutaminase (Gls), nuclear gene encoding mitochondrial protein | **0.62** | 0.009 |
| Csnk2a1 | casein kinase 2, alpha 1 polypeptide | **0.72** | 0.022 |
| Nme3 | non-metastatic cells 3, protein expressed in | **1.33** | 0.022 |
| Anxa1 | annexin A1 | **1.37** | 0.024 |
| Mboat1 | membrane bound O-acyltransferase domain containing 1 | **1.44** | 0.018 |
| Begain | brain-enriched guanylate kinase-associated | **1.45** | 0.018 |
| Gng10 | guanine nucleotide binding protein (G protein), gamma 10 | **1.47** | 0.018 |
| Gatm | glycine amidinotransferase (L-arginine:glycine amidinotransferase) | **1.52** | 0.012 |
| Akr1c14 | aldo-keto reductase family 1, member C14 | **1.56** | 0.013 |
| Bdh1 | 3-hydroxybutyrate dehydrogenase, type 1 | **1.68** | 0.020 |
|  |  |  |  |
| *Unclassified* | |  |  |
| Scgb1c1 | secretoglobin, family 1C, member 1 | **0.60** | 0.005 |
| Heatr5b | HEAT repeat containing 5B | **0.62** | 0.014 |
| X05545 | mRNA fragment for gag related peptide | **0.63** | 0.017 |
| Homer1 | homer homolog 1 | **0.64** | 0.012 |
| Tbx20 | T-box 20 | **0.69** | 0.020 |
| Fam160a1 | family with sequence similarity 160, member A1 | **0.70** | 0.024 |
| Ythdf3 | YTH domain family 3 | **0.70** | 0.014 |
| Fignl1 | fidgetin-like 1 | **1.38** | 0.018 |
| Igfbp4 | insulin-like growth factor binding protein 4 | **1.48** | 0.017 |
| Pqlc3 | PQ loop repeat containing 3 | **1.57** | 0.018 |
| Morn4 | MORN repeat containing 4 | **1.62** | 0.005 |
| Fam81a | family with sequence similarity 81, member A | **1.66** | 0.005 |
| Crocc | ciliary rootlet coiled-coil, rootletin | **1.69** | 0.012 |
